# Cognitive aging and hearing acuity: modeling spoken language comprehension

**DOI:** 10.3389/fpsyg.2015.00684

**Published:** 2015-06-11

**Authors:** Arthur Wingfield, Nicole M. Amichetti, Amanda Lash

**Affiliations:** Volen National Center for Complex Systems, Brandeis University, Waltham, MA, USA

**Keywords:** speech recognition, working memory, inhibition, sentence comprehension, ELU model

## Abstract

The comprehension of spoken language has been characterized by a number of “local” theories that have focused on specific aspects of the task: models of word recognition, models of selective attention, accounts of thematic role assignment at the sentence level, and so forth. The ease of language understanding (ELU) model (Rönnberg et al., 2013) stands as one of the few attempts to offer a fully encompassing framework for language understanding. In this paper we discuss interactions between perceptual, linguistic, and cognitive factors in spoken language understanding. Central to our presentation is an examination of aspects of the ELU model that apply especially to spoken language comprehension in adult aging, where speed of processing, working memory capacity, and hearing acuity are often compromised. We discuss, in relation to the ELU model, conceptions of working memory and its capacity limitations, the use of linguistic context to aid in speech recognition and the importance of inhibitory control, and language comprehension at the sentence level. Throughout this paper we offer a constructive look at the ELU model; where it is strong and where there are gaps to be filled.

## Introduction

Raymond Carhart has been credited with coining the term “audiology” (an interesting mix of Latin and Greek roots), and offering the first formal course with that name at Northwestern University in 1946. In its early beginnings the issue of cognition played no role in research or teaching on hearing loss. In Newby’s (1958) then-classic text in audiology, for example, the focus was squarely on peripheral hearing loss; any issues related to the pathways from the brain stem to and including the cortex was cited as the domain of neurology (Newby, 1958, pp. 53–55). Indeed, beyond supplying a definition of presbycusis as an age-related hearing loss, adult aging received no additional attention.

It is now well recognized that older adults’ success in speech recognition, especially under difficult listening conditions, will be affected by cognitive factors: either in a positive way through support from linguistic context, or in a negative way where performance can be constrained by limitations in working memory and executive resources (van Rooij and Plomp, 1992; Humes, 1996; Gordon-Salant and Fitzgibbons, 1997; Wingfield and Tun, 2001; Pichora-Fuller, 2003). Just as audiology has begun to recognize that cognitive factors may play a role in performance, so cognitive psychologists engaged in research on language comprehension in older adults are beginning to recognize that the full picture of language comprehension cannot be understood without attending to the auditory declines that are common in normal aging. The joining of these two areas of expertise has seen a dramatic increase, giving rise to such terms as “cognitive hearing science” (Arlinger et al., 2009) and “ cognitive audiology ” (Jerger, cited in Fabry, 2011, p. 20). The introduction of these terms reflects an increasing emphasis on the importance of taking into account how cognitive processes interact with hearing acuity in communicative behavior and remediation strategies to deal with hearing loss.

The broad sweep of issues underlying sensory-cognitive interactions in the perception and comprehension of speech raises the need for a unifying framework to guide present and near-future research. The *Ease of Language Understanding* (ELU) model (Rönnberg, 2003; Rönnberg et al., 2008, 2013) stands as such attempt. In this article we examine aspects of the ELU model that apply especially to spoken language comprehension in adult aging, where speed of processing (Salthouse, 1996), working memory capacity (Salthouse, 1994), and hearing acuity (Lethbridge-Ceijku et al., 2004) are often compromised. Throughout, we hope to offer a constructive look at the ELU model; where it is strong and where there are gaps to be filled. In so doing we use this discussion as a vehicle to examine interactions of perceptual, linguistic, and cognitive factors in spoken language understanding.

## The ELU Model: A Brief Summary

The ELU model has developed from its original version (Rönnberg, 2003) to the more inclusive model as it is presented today (Rönnberg et al., 2013). The 2003 paper presents a basic framework along with a formulation to capture four parameters of spoken language understanding: (1) accuracy and features of syllable representations; (2) the speed of access to long-term memory (LTM); (3) the level of mismatch between the stimulus input and the corresponding phonology represented in the mental lexicon; and (4) the processing efficacy and storage capacity of working memory. This initial model assumed an interaction between the quality of the sensory input, information available in LTM, and the utilization of working memory. Together these would determine the ease with which language can be comprehended under difficult listening conditions. An important element in this initial presentation was a model assumption that phonological and lexical access are automatic (implicit) as long as no mismatch occurs between the sensory input and stored lexical representations. When a mismatch occurs processing becomes explicit, represented by employment of supportive context and engagement of working memory resources. This early foundation thus assumed a fundamental division between implicit and explicit components in speech understanding.

The 2013 version (Rönnberg et al., 2013) became more nuanced and more specific. In the former case it was now argued that implicit and explicit processing may operate on the interaction of phonology and semantics in parallel. As such, long-term memory (LTM) can be used either explicitly (a slow process) or implicitly (a rapid process) for understanding a spoken message. There was also an increasing attempt to say how working memory capacity relates to attention, short-term storage, inhibition, episodic LTM, and listening effort. In addition, the model in 2013 distinguishes between types of LTM (episodic and semantic) and how and when these memory systems are accessed at different stages of understanding. Rönnberg et al.’s (2008) version implied a solely feed-forward system, with the rapid and automatic multimodal binding of phonology taking place in an episodic buffer through implicit processing that matches inputs with stored representations in the mental lexicon. The 2013 version now recognizes the involvement of continuous feedback with both predictive and post-dictive (backward) feedback loops. This latter presumption is necessary given findings such as, for example, the demonstration that the perception of sub-lexical sounds are influenced by top-down word knowledge (Samuels, 2001).

Finally, in Rönnberg et al. (2013) the ELU model has been broadened to include multimodal integration in the form of visual information from seeing a talker’s articulatory movements, processed in a modality-general limited capacity working memory system. In this latter regard there is certainly ample evidence for multimodal integration beginning with Sumby and Pollack’s (1954) demonstration that people perceive speech in noise better when they can see the speaker’s face. Access to such visual information can also be advantageous for older adults (Sommers et al., 2005; Feld and Sommers, 2009). With these recent revisions, the ELU model sets up a new line of predictions. Many of these predictions relate to the effects of different signal qualities, the type and modality of the inputs (hearing, vision, and sign language), and the relationship of working memory capacity to different encoding operations and other memory systems.

Although the ELU model has become more inclusive, there are aspects of language processing that remain underrepresented in model. We address several of these issues below. In so doing we place special emphasis on spoken language understanding by older adults following typical age-related changes in cognitive efficiency and hearing acuity. As we shall see, the cognitive literature, upon the ELU model should rely, remains unsettled on many critical issues. These issues also form a part of our discussion.

## Conceptions and Control Functions in Working Memory and its Capacity

As we have noted above, working memory plays a central role in the ELU model, where it is seen as carrying a number of cognitive functions relevant to language understanding. Most conceptions of working memory in the cognitive literature have in one way or another postulated a trade-off between processing and storage, whether conceived in terms of a shared general resource (Just and Carpenter, 1992; Carpenter et al., 1994), or a limited-capacity central executive (Baddeley, 1996; Logie, 2011). Mechanisms that have been proposed to underlie the limited capacity of working memory have included time-based models in which switching attention from processing to storage or updating and refreshing the memory trace are constrained by the time parameters of these processes (Barrouillet et al., 2004, 2012). In this latter regard descriptions of working memory and executive function begin to merge, with these terms often used along with the even more general term, “resources” (often, without distinction, referred to as attentional resources, processing resources, or cognitive resources).

A model that focuses on language understanding under adverse listening conditions would benefit greatly if it could rest on settled conceptions of working memory and executive function in the general cognitive literature. As yet such a simple consensus has yet to emerge. It might be helpful to adopt McCabe et al.’s (2010) characterization of working memory as focusing on the ability to store and manipulate information, and executive function as focusing on goal-directed behavior, monitoring and updating performance, set shifting, and inhibition (cf. Hasher and Zacks, 1988; Hasher et al., 1991; Cowan, 1999; Engle, 2002; Fisk and Sharp, 2004; Bopp and Verhaeghen, 2005; Logie, 2011), albeit with each containing elements of the other and all of these abilities associated with activity in prefrontal cortex (McCabe et al., 2010).

In its current version the ELU model cites the importance of inhibition and executive function in speech processing, but the relationship between these functions and working memory are as yet not clearly articulated within the model (Rönnberg et al., 2013, p. 10). The challenge in doing so is highlighted in McCabe et al. (2010) who report a strong correlation between tests of working memory capacity and those purported to test executive functioning (*r* = 0.97), with only processing speed showing independence. Although there is agreement that working memory capacity is limited, and more limited in older relative to younger adults (Salthouse, 1994, 1996; Salthouse et al., 2003), there is no uniform agreement within the cognitive aging literature on the mechanisms that underlie this limitation.

Our own view is closely aligned with the postulate that working memory capacity is determined by how well one can focus attention (Engle et al., 1999; Engle and Kane, 2004). A case in point is Cowan’s (1999) Embedded-Process model that sees working memory as an activated subset of information within LTM. The source of the well-known capacity limitation in working memory is seen as due to the limited capacity of attentional focus that operates on the activated areas within LTM (Cowan, 1999). As such, the capacity of working memory arises from both a time limit on activation of items in memory, unless refreshed, and a limit on attentional capacity in terms of the number of items that can be concurrently activated (Cowan, 2005). What we describe here is a process-based view of working memory and working memory capacity that allows concurrent activation of representationally distributed information, a potential mechanistic account for the modality-general aspects of working memory postulated in the ELU model.

### Control Functions in Working Memory

The emphasis in the ELU model is on communication, which sets it apart from many extant models of speech recognition and language understanding that focus more narrowly on specific processes and in many cases do not address how the systems operate under adverse listening conditions. Considerable research has shown that the perceptual effort attendant to poor listening conditions has a negative impact on recall of speech materials (Rabbitt, 1968, 1991; Pichora-Fuller et al., 1995; Surprenant, 1999, 2007; Wingfield et al., 2005) and comprehension of sentences that express their meaning with non-canonical word orders typical of syntactically complex speech, with this latter effect compounded by effects of age, hearing acuity, and rapid speech rates (Wingfield et al., 2006).

**FIGURE 1:**
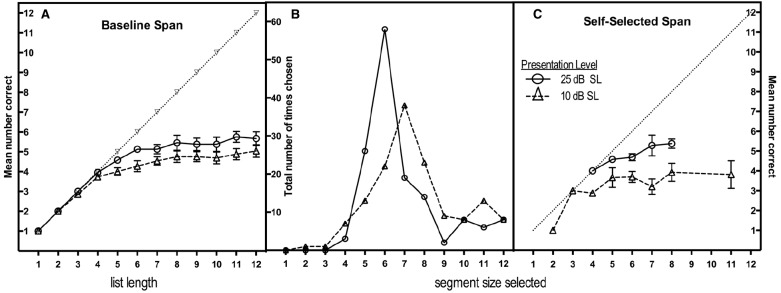
**Mean number of words correctly recalled as a function of the number of words presented (baseline span) for words presented at either 25 dB SL or 10 dB SL (A); the distribution of segment sizes selected in the interruption-and-recall (IAR) condition at the two sound levels (B); and the number of words recalled as a function of the number of words selected in the IAR condition at the two presentation levels (C).** Error bars in left and right panels represent one standard error. (From **Figure 2** in Amichetti et al., 2013, Copyright 2013 by the Psychonomic Society. Reprinted with permission.)

In the ELU model the degree of effort engendered by task difficulty affects the degree to which explicit processing will be engaged. Among such explicit processes must be an ability to monitor the ongoing capacity of working memory as speech arrives in real time. Figure 1 shows data taken from our laboratory in which we probed the effect of listening effort on the ability to monitor the capacity of working memory as speech is arriving in real time. For this purpose we used an *interruption-and-recall* (IAR) paradigm in which participants listen to a string of recorded words with instructions to interrupt the input when they believe they have heard the maximum number of words that will allow for perfect recall of what has been heard. Germaine to our present interests, the word-lists were presented at one of two sound levels: at 25 dB SL to represent listening ease, and 10 dB SL to represent effortful listening. The participants were young adults with age-normal hearing (Amichetti et al., 2013, Experiment 2).

Figure 1A shows the mean number of words correctly recalled in a simple baseline span task in which listeners heard lists varying in length from one to 12 items for immediate recall. It can be seen that for list lengths of up to three words recall is at ceiling, and at near-ceiling for a four-item list length at both intensity levels, thus confirming the audibility of the stimuli at the two sound levels. Beyond a four-item list, additional stimulus items yield progressively smaller recall gains that never peak beyond means of 5.8 items for the 25-dB SL lists and 4.3 items for the 10-dB SL lists. This small but significant difference affirms the above-cited negative effect of effortful listening on recall.

Figures 1B,C are of greater interest as they show what happened when participants heard supra-span lists with instructions to interrupt the word-lists with a keypress when they believed they had heard the maximum number of words that they could recall with perfect accuracy. The middle panel shows the distribution of segment sizes participants selected for recall for the 25-dB SL and 10-dB SL presentation levels in this IAR condition. One can see a shift in the peaks of the two distributions, from a modal self-selected segment length of six words for lists at the louder 25-dB SL level, to seven words, at the 10-dB SL level. Specifically, at 25 dB SL the modal segment size of six words was close to the mean for accurate item recall of 5.8 words in the baseline span condition at that sound level shown in the left panel, suggesting a good ability to calibrate segment size selections with actual memory span. By contrast, in the effortful listening condition, listeners appeared to lose this close calibration. That is, a reduced memory span for accurate recall of 4.3 words for 10 dB SL lists in the baseline condition was not accompanied by listeners adaptively taking shorter segment sizes for recall in the IAR condition.

The right panel shows the number of words recalled in the IAR conditions for list lengths that had more than 10 examples on which to base a mean. The dual-task nature of the IAR condition (the listener must make continuous capacity judgments while holding what has been heard to that point in memory) reflects a greater cognitive load than in baseline span task. As would be expected if listening effort draws on already strained resources in the IAR task, while for the 25-dB SL presentations the IAR-produced spans are similar to baseline spans at 25 dB SL, recall accuracy for the IAR spans at the more effortful 10 dB SL level were reduced relative to the corresponding baseline span presented at 10 dB SL.

As we have noted, the ELU model asserts that a degraded (perceptually effortful) signal leads to a shift from automatic to controlled processing with an engagement of working memory resources. We show with the above data that this control itself may be affected by the necessity to process a low-quality signal. In part the lower sound level may have slowed the stimulus encoding, resulting in an overlap in time in which the cognitive system is concurrently conducting perceptual and encoding operations on one stimulus as another is arriving (Miller and Wingfield, 2010; Piquado et al., 2010). It is also possible that a reduced stimulus intensity may truncate the duration of an already rapidly fading echoic trace (Baldwin, 2007; Baldwin and Ash, 2011).

This control function in working memory may be obscured in natural speech if listeners are allowed to periodically interrupt a spoken narrative to give themselves time to process what they have heard before the arrival of yet more information. In this case both young and older adults tend to interrupt the speech input at major linguistic clauses and sentence boundaries rather than after a set number of words (Wingfield and Lindfield, 1995; Piquado et al., 2012; see also Wingfield et al., 1999; Fallon et al., 2006). Importantly, such findings are indicative of listeners’ access to syntactic and semantic knowledge as the speech is being heard, and hence being involved in very early stage processing. We will address the implications of early access in several places in the following discussion.

## The Implicit versus Explicit Distinction

Fundamental to the ELU model is the position that when speech quality is good, with a clear match between acoustic input and its corresponding phonological representation in LTM, lexical recognition will be automatic (“implicit”). That is, lexical access will be rapid, resource-free, and will not require access to top-down information such as linguistic or semantic context. When the input quality is poor, whether due to external factors such as background noise, or internal factors such as hearing loss or a distorted phonological representation in LTM consequent to a long-term hearing impairment, the degraded information can be supplemented by linguistic or real-world knowledge, a process that requires explicit or “effortful conscious processing” (Mishra et al., 2013, p. 2).

Use of the terms *implicit* and *explicit* processing in the ELU model resonate with the early (LaBerge and Samuels, 1974; Shiffrin and Schneider, 1977), but still often used distinction in the cognitive literature between *automatic* versus *controlled processes*. In the context of speech perception, automatic processes emphasize bottom-up, stimulus-driven processing that is rapid, obligatory, and demanding few if any resources. By contrast, controlled processes tend to be top-down, voluntary, and to one degree or another resource-demanding (Pashler et al., 2001). They are also assumed to require some level of awareness (LaBerge and Samuels, 1974; Posner and Snyder, 1975; Shiffrin and Schneider, 1977; Flores d’Arcais, 1987). All of these attributes fit squarely with the characterization of implicit and explicit processing as represented in the ELU model.

Although early-stage perception is often considered to be automatic, arguments have been offered for cognitive and attentional control operating at the earliest stages of input processing of speech (Nusbaum and Magnuson, 1997; Heald and Nusbaum, 2014). It should also be recognized that a system that appears to be resource-free could require resources but not those shared with other processes. This exact position was taken by Caplan and Waters (1999) who argued that on-line syntactic operations are conducted by sentence-specific resources not measured by traditional working memory tasks such as the Daneman and Carpenter (1980) readings span task or its several variants. They suggest that the appearance of effects working memory limitations on sentence processing represent post-interpretive processes rather than on initial syntactic parsing. Our present focus, however, is the specific assertion in the ELU model that when there is degraded input perceptual operations will shift from automatic to controlled processing, with the latter increasing the drain on working memory resources (Rönnberg et al., 2013).

Proposals of binary, either-or process distinctions have been a hallmark of early theory development in cognitive psychology such as distinctions drawn between semantic versus episodic memory (Tulving, 1972), procedural versus non-procedural learning (Squire, 1994), implicit versus explicit memory in reference to priming studies (Schacter, 1987), and so forth. In each case subsequent studies have shown none of these proposed distinctions to be process pure. In a similar way, the distinction between automatic (implicit) versus controlled (explicit) processes can best be seen as two ends of a continuum and a matter of degree rather than the sharp contrast current in the ELU model.

Although drawing a distinction between implicit and explicit processes, Rönnberg et al. (2013) note that the extent to which explicit or implicit processing may be employed can vary over the course of a single task, with the ratio changing from moment to moment during a conversation depending on signal quality and speech content (see also Rönnberg et al., 2010).

It is the case that the automatic versus controlled distinction retains descriptive utility (Birnboim, 2003; Schneider and Chen, 2003), but only insofar as one thinks of some operations being potentially “more automatic” than others in a relative or graded sense (Chun et al., 2011).

## The Match versus Mismatch Distinction

The match versus mismatch distinction highlighted in the ELU model may be accepted as an idealized principle, although such a distinction should be treated with caution. This is so because there is rarely a perfect match between a phonological input and the phonological representation of an item in the mental lexicon. This is due to the variability in the way words and their sub-lexical elements are articulated from speaker to speaker, and effects of syllabic context within a single speaker (Liberman et al., 1967; Mullennix et al., 1989).

At the more cognitive level, analyses of natural speech show that speakers tend spontaneously to employ a *functional adaptation* in their production. That is, we tend to articulate more clearly words that cannot be easily inferred from context, and to articulate less clearly those that can (Hunnicutt, 1985; Lindblom et al., 1992). It is not assumed that these dynamic adjustments are consciously applied by the speaker, any more than we assume that listeners are necessarily consciously aware of using acoustic and linguistic context in their perceptual operations.

Because of this functional adaptation, what one might call an articulatory *principle of least effort*, words are often under-articulated when they can be predicted from the context, and many words would be unintelligible were it not for the phonemic and linguistic context in which they are ordinarily heard (Lieberman, 1963; Pollack and Pickett, 1963; Grosjean, 1985; Wingfield et al., 1994). Because of this variability perfect-match template matching models of perception must be an ineffective account of perceptual identification. To the extent that the ELU model presumes a perfect or near perfect match between phonological inputs and stored counterparts in LTM as the default condition with natural speech, this would be out of tune with these data. It should be noted that although the early Rönnberg (2003) formulation implied a stark contrast between a perfect match versus one that requires top-down support, the current model version sees word recognition in terms of a threshold function affected by phonological and semantic attributes (Rönnberg et al., 2013). This question relates to broader issues in the role of linguistic context in speech recognition and comprehension.

## The Role of Context

A common view in speech recognition is that questions related to effects of context should be framed in terms of top-down effects operating on initially stimulus-driven perceptual processes. The ELU model is in general accord with this principle, although an apparently conflicting observation appears in the suggestion in Rönnberg et al. (2013) that if a sentence context is sufficiently predictive, a target word might be activated even with minimal phonological input (Rönnberg et al., 2013). This presumption, although consistent with everyday experience, would not seem to follow at first look from the precepts of the ELU model. It would follow, however, from a number of extant models of word recognition.

Most models of word recognition, to include the ELU model, assume a reciprocal balance between bottom-up information determined by the clarity of the speech signal and top-down information supplied by a system of linguistic knowledge (e.g., Morton, 1969, 1979; McClelland and Elman, 1986; Marslen-Wilson, 1987). It is the compensatory availability of preserved linguistic knowledge and the procedural rules for its use that accounts for the general effectiveness of speech comprehension in adult aging in spite of cognitive and sensory declines (Wingfield et al., 1991; Pichora-Fuller et al., 1995; Wingfield and Stine-Morrow, 2000; Pichora-Fuller, 2003). Although these principles are embodied within the broad outlines of the ELU model, questions remain as to whether context comes into play before, during, or after the acoustic representation of a word unfolds in time.

A model that assumes that context activates lexical possibilities before a stimulus word is heard was embodied in one of the earliest interactive models: the so-called “logogen” model that also went through a period of development (Morton, 1964a,b, 1969, 1979). Morton postulated a “dictionary” of “units” (later re-named “logogens”), with each unit corresponding to a word represented in LTM. When the level of activation of a logogen exceeds a critical level, the unit “fires,” and the corresponding word is available as a response.

In this model each unit has a resting potential, or base level of activation, determined by the relative frequency with which the unit has fired in the past. This is reflected behaviorally in the *word frequency effect*, in which words that have a high frequency of occurrence in the language are recognized faster or with less stimulus information than low-frequency words (Howes, 1957; Grosjean, 1980). Following the firing of a unit its resting level of activation increases sharply, resulting in recency or repetition priming, and then decays slowly. Through direct connections with other units, the activation of any given unit adds to the level of activation of all associated units, whether this association is semantic, categorical, or based on shared attributes.

In operation, a sensory input would be coded in terms of the presence of detected phonological features, the presence of which would simultaneously increase the level of activation of all units sharing these phonological features. Thus, the unit sharing the greatest number of features with the presented stimulus would receive the greatest increase in its level of activation. It can be seen from this formulation that the amount of stimulus information required for a unit to exceed its critical level and “fire,” would be lower either when there is already a high level of residual activation (the word frequency effect), when the level of activation has been temporarily raised by a recent firing of the unit (recency priming), or by the firing of an associated unit or units (an effect of context).

Within the Logogen model, a highly constraining linguistic or environmental context that increases the likelihood of occurrence of a stimulus word will increase the level of activation of that item in the mental lexicon, thus priming the entry even before the stimulus is actually encountered. The higher the level of activation, the less stimulus information will be required for recognition of the target word. Activation due to contextual expectancy would thus override units’ initial resting potentials initially determined by their relative frequency of occurrence in the language, and hence, their likelihood of re-occurrence. A constraining linguistic or environmental context would also override other factors known to affect the intelligibility of individual words, such as the detrimental effect of a large number of words that share initial or overall phonology with the target word (cf. Tyler, 1984; Wayland et al., 1989; Wingfield et al., 1997; Luce and Pisoni, 1998). These general principles have been embodied in a number of models, to include TRACE, a computational model in which the above factors, operating in parallel, can be implemented by transient weighting factors (McClelland and Elman, 1986).

A correlate of Morton’s model is that if the level of activation of a lexical unit is sufficiently raised due to a high probability of it being encountered, a lexical unit may “fire” in the absence of objective stimulus information. It can be seen that Morton’s logogen model and others like it offer a mechanistic account noted by Rönnberg et al. (2013) that if a sentence context is sufficiently predictive, a target word might be activated even with minimal phonological input. This principle of an inverse relationship between the *a priori* probability of a word and the amount of phonological information needed for its recognition is a well established finding in the literature for both spoken and written words and for both young and older adults (Black, 1952; Bruce, 1958; Morton, 1964a,b; Cohen and Faulkner, 1983; Madden, 1988). It should be pointed out, of course, that the more likely scenario following the same principle is the misidentification of an indistinct word as a word with a similar sound that is a closer fit to a semantic context (Rogers et al., 2012). Either case, however, would necessitate a closer look within the ELU model at whether context raises lexical activation before (Morton, 1969), during (Marslen-Wilson and Zwitserlood, 1989), or after (Swinney, 1979) the word unfolds in time.

In contrast with models that assume that linguistic context raises target activation even prior to acoustic input, we have seen that a basic tenet of the ELU model is that an acoustically clear stimulus with a correspondingly rich mental representation results in automatic (implicit) lexical access; a rapid, obligatory, resource-free process. In the model context comes into play only when poor stimulus quality does not allow an immediate match at which point context “kicks in.” The process being described is suggestive of early modular models of lexical access such as Forster’s (1976; 1981) argument for autonomous lexical access: a self-contained modular system, with restricted access to information. Such an “informationally encapsulated” (context-free) process fit within Fodor’s (1983) broader argument for modularity within cognitive domains and processes.

The positive influence of a constraining sentence context or other sources of semantic priming on the accuracy or speed of lexical access (e.g., Holcomb and Neville, 1990) appears as inconsistent with the postulate of a context-impenetrable modular view of lexical access. This issue is not easily settled in spite of a history of creative experiments intended to determine whether the facilitation observed with a constraining sentence context reflect a true access effect (cf., Swinney, 1979; Seidenberg et al., 1982, 1984; Stanovich and West, 1983).

The issue is whether the well-documented effects of expectancy on ease of lexical access, and especially the suggestion that a sufficiently strong expectation can activate a lexical entry in the absence of sensory input, is most compatible with a pre-lexical (e.g., Morton, 1969) or a post-lexical (e.g., Forster, 1981) effect. Our reading of the ELU model appears to favor both positions, an issue that would need to be reconciled as the model develops in detail.

Before leaving this issue, we might also suggest that a complete model for word recognition should include not only the level of activation of a lexical entry as determined by contextual expectancy and the goodness of fit with the stimulus, but also on the individual’s acceptance criterion level. This flexible criterion level would be determined by such factors as the priority given to speed versus accuracy (Wagenmakers et al., 2008) or the reward for a correct recognition versus the negative consequences of making an erroneous identification (Green and Swets, 1966). This position thus adds motivational state to the quality of the sensory input and the sensory capacities of the listener.

## Age and Inhibition in Word Recognition: The Role of Working Memory

Benichov et al. (2012) examined ease of recognition of sentence-final words heard in noise with participants aged 19–89 years, with levels of hearing acuity ranging from normal hearing to mild-to-moderate hearing loss. Regression analyses showed that hearing acuity, although a predictor of the signal to noise ratio necessary to correctly recognize a word in the absence of a constraining linguistic context, dropped away as a significant contributor to recognition of sentence-final words by the time the linguistic context was strongly predictive. By contrast, a cognitive composite of individuals’ episodic memory, working memory, and processing speed accounted for a significant amount of the variance in word recognition for words heard in a neutral context and for all degrees of contextual constraint examined. (The contextual probability of the target words was taken from published “cloze” norms, which report the percentage of participants who give particular words when asked to complete sentence stems with the final word missing.)

One likely candidate for the role that working memory capacity may play in word recognition was revealed in a study by Lash et al. (2013) who examined effects of age, hearing acuity, and expectations for the occurrence of a word based on a linguistic context. Importantly, the study also examined the effects of competition from other words that might also fit the semantic contexts. Lash et al. (2013) used the technique of *word-onset gating*, in which a listener is presented with an increasing amount of a word’s onset duration until the word can be correctly identified (Grosjean, 1980, 1996). When a linguistic context is absent, word recognition is affected by the number of words that share the initial sounds with the target word (Tyler, 1984; Wayland et al., 1989), further limited by words that share syllabic stress (Wingfield et al., 1997; Lindfield et al., 1999; see also Wingfield et al., 1990).

A major focus of the Lash et al. (2013) study was the effect of a linguistic context on word recognition that, as we have previously indicated, will override such factors as word frequency or the number (“density”) of phonological competitors as determinants of word recognition. A critical feature of published cloze norms (e.g., Lahar et al., 2004), however, is that when participants have been asked to complete sentence stems, also reported is the full range of responses given by each of the participants, and the number of participants giving these alternative responses. These data allow one to estimate not only the expectancy of a sentence-final word based on the transitional probability of that word in the sentence context, but also the uncertainty (*entropy*) implied by the number, and probability distribution, of alternative responses that also might be implied by the context. Lash et al. (2013) found that while both young and older adults’ word recognition benefitted from a sentence context that increased word expectancy, a differentially negative effect of the presence of strong competitor responses was found for older adults independent of hearing acuity.

This latter finding is consistent with Sommers and Danielson’s (1999) proposition that older adults have greater difficulty than their young adult counterparts in inhibiting non-target responses. In Sommers and Danielson’s (1999) case the competition came from the presence of a larger number of phonological “neighbors” of target words. The present case differed only in that response competition came from the distribution of words that also shared a contextual fit with a semantic context. Such results would be expected from arguments that older adults have a general inhibition deficit (Hasher and Zacks, 1988), that in this case, would interfere with word recognition.

A subsequent study by Lash and Wingfield (2014) directly examined working memory capacity and effectiveness of inhibition in word recognition as would be predicted from observations present in the current version of the ELU model. This study was based on the finding that gradually increasing the clarity of a stimulus until it can be correctly identified retards its recognition relative to when a stimulus is presented just once, even at a level of clarity below that needed for recognition using an ascending presentation. This finding, observed originally for degraded visual stimuli, has been interpreted as reflecting the negative effect of interference from incorrect identification hypotheses formed during the incremental presentations that would not be present with a single presentation (Bruner and Potter, 1964; Snodgrass and Hirshman, 1991; Luo and Snodgrass, 1994).

Lash and Wingfield (2014) conducted an analogous study for spoken words using word-onset gating with older adults (*M* = 75 years) with good hearing acuity (PTA < 25 dB HL) and an age-matched group with a mild-to-moderate hearing loss. A group of young adults with normal hearing acuity was also included for comparison. For each individual we determined the word-onset gate size that allowed the participant to recognize correctly 40 to 60% of target words when they were presented successively with increasing onset durations (an *ascending presentation*). We also determined for each individual the recognition accuracy level for comparable words presented just once (a *fixed presentation*) with the same gate size that yielded the 40 to 60% correct recognition with an ascending presentation. The size of the interference effect from ascending presentations would be indexed by the difference between word identification rates under the two presentation conditions. The question was whether individual differences in working memory capacity might predict one’s ability to inhibit interference from false identification hypotheses presumed to be formed in the course of the incrementally larger and larger word onset durations represented in the ascending presentation condition (e.g., Snodgrass and Hirshman, 1991; Luo and Snodgrass, 1994).

As might be expected from age and inhibition arguments, the older adults in the study showed a larger interference effect from ascending presentations than the young adults. Germaine to our present question, a follow-up regression analysis revealed that participants’ reading spans, taken as a measure of working memory capacity (Daneman and Carpenter, 1980; McCabe et al., 2010), contributed significantly to the size of the interference effect (see Lash and Wingfield, 2014, for full details). The reading span test, which we discuss in a subsequent section, was used rather than a listening span version (e.g., Wingfield et al., 1988) to avoid a potential confound with hearing acuity.

**FIGURE 2:**
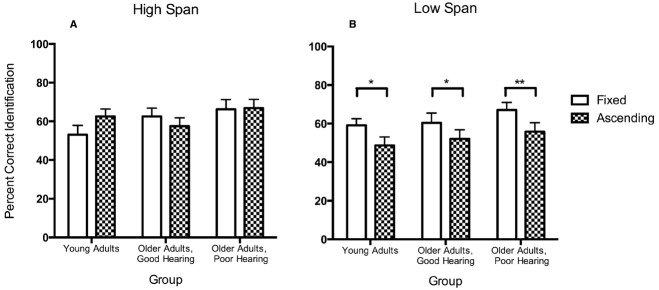
**Percentage of words correctly identified with the same onset gate size when stimuli were presented under fixed versus ascending procedures for young adults with age-normal hearing acuity and older adults with good hearing acuity or a mild-to-moderate hearing loss.** Panel **(A)** shows participants with high working memory spans. Panel **(B)** shows these data for participants with lower working memory spans. Error bars represent one standard error. (Data from Lash and Wingfield, 2014, Psychology and Aging, Viol. 29.) *p < 0.05, **p < 0.01.

This effect of working memory span on the effectiveness of inhibition can be illustrated most clearly in Figure 2 in which we have taken data from Lash and Wingfield (2014) and have plotted the percentage of correct identifications for the same gate size when words were presented in the fixed versus the ascending presentation conditions separated by participants’ working memory span. A participant was considered to have a high working memory span (left panel) if they scored greater than one standard deviation above the mean for their age cohort determined by McCabe et al. (2010), or a low span if they did not (right panel). These data are based on a subset of participants from Lash and Wingfield (2014) where high and low span participants within each participant group were equal in number and matched for age.

Although for the high span participants some variability appears in the difference between identification scores for the fixed versus ascending presentation conditions, especially for the young adults, none of these differences reached significance. By contrast, the lower span participants in each of the three participant groups consistently show a significant interference effect even after adjusting for differences in baseline recognition accuracy.

These data can thus be taken to offer empirical support for the suggestion in Rönnberg et al. (2013) that working memory capacity may affect the efficiency of inhibitory processes (see also Sörqvist et al., 2012). It should be noted, however, that a relationship between working memory capacity and effectiveness of inhibition leaves open the direction of causality. Indeed, an influential argument has been made that it is a failure of the ability to inhibit off-target interference that may determine the size of one’s working memory capacity (Hasher and Zacks, 1988; Hasher et al., 1991). We will have more to say on this topic in the following section.

## Input Challenge at the Sentence Level: Deep versus Shallow Processing

A premise of the ELU model is that a perceptual mismatch due to a poor quality stimulus causes a shift from implicit (automatic) to explicit (controlled) processing where support from linguistic or environmental context are brought into play through involvement of working memory. As outlined in the model, this shift will slow processing but hopefully lead to a successful solution. Because syntactic resolution of a sentence is arguably a precursor to determination of sentence meaning, this would imply that, when speech quality is poor, listeners will engage in an especially detailed and explicit syntactic analysis. Rönnberg et al. (2013), however, offer a qualification: when placed under time pressure, and if the listener is willing to accept the gist of the message, such a close analysis might not take place (Rönnberg et al., 2013, p. 10).

There is no doubt that this latter point is true, both intuitively and empirically. We would suggest, however, that in natural language comprehension such gist processing may be the rule rather than the exception. This would be so since in listening to spoken discourse one is almost always under time pressure due to the rapidity of natural speech and the transient nature of the speech signal. Ordinary speech rates average between 140 to 180 words per minute, and can often reach 210 words per minute as, for example, with a radio of TV newsreader working from a prepared script (Stine et al., 1990).

Although in many cases a complete syntactic analysis may be conducted as a precursor to determining a sentence meaning, there is considerable evidence that listeners often, perhaps more often, take processing short-cuts, sampling key words and using plausibility to understand the meaning of an utterance. Because we live in a plausible world this strategy will in most cases yield rapid and successful comprehension, albeit with comprehension errors should one encounter a sentence with an unexpected or implausible meaning.

Analyses of everyday discourse show that most of our sentences, when they are in fact grammatical, tend to have meaning expressed in a relatively simple noun-verb-noun canonical word order with the first word representing the agent or source of the action (Goldman-Eisler, 1968). Thus, so long as the syntax is represented by canonical word order and the meaning of a sentence is plausible, a gist analysis will most often yield a correct understanding. This strategy goes unnoticed because it invariably works; it is revealed, however, when comprehension fails. In such cases listeners “mishear” a sentence as if it were sensible, such as the sentence, “The teenager that the miniskirt wore horrified the mother” (Stromswold et al., 1996). Examination of individuals’ comprehension of such sentences have shown that comprehension errors frequently occur, suggesting the absence of a full syntactic analysis of a sentence input in favor of sampling key words, assuming that the word order represents the meaning in a canonical form, and that the semantic relations being expressed in the sentence are plausible (Fillenbaum, 1974; Sanford and Sturt, 2002).

Ferreira (2003) has formalized these notions, suggesting that heuristic short-cuts may be taken by all listeners, by-passing a full syntactic analysis but instead using word-order and plausibility as a rapid first-pass comprehension strategy (Ferreira et al., 2002; Ferreira, 2003; Ferreira and Patson, 2007). As Ferreira et al. (2002) have argued, it should not be assumed that all relevant information from a detailed and time-consuming lexical and syntactic analysis will be used in everyday comprehension. Sanford and Sturt (2002), from the perspective of computational linguistics, come to a similar conclusion. That is, to use Ferreira and Patson’s (2007) words, sentence processing is as often as not conducted at a level of analysis that is “good enough” for comprehension. As we have argued above, this processing strategy will yield the right answer more often than not. It is consistent with the slowed processing and limited working memory capacity of older adults that Christianson et al. (2006) have argued that a “good enough” processing heuristic may be even more common in the elderly.

## Working Memory and Language Comprehension

There are a variety of working memory measures in the literature designed to capture operational capacity. Important among them is the reading span task introduced by Daneman and Carpenter (1980), that focuses more specifically on verbal working memory (Carpenter et al., 1994). It is a version of this reading span task that serves as the preferred measure of working memory in the ELU-related studies conducted by the Rönnberg group.

The reading span (or listening span) task requires the listener to read (or listen to) a series of sentences and, to insure the sentences are being comprehended, to state after each sentence whether it is true or false, or in some variants, whether the meaning of the sentence is plausible or implausible. After a set of sentences is finished the reader (or listener) must recall the final word of each sentence, or he or she receives a signal to recall either the last word or the first word of each of the sentences. The span is taken as the number of sentences that allow accurate recall of the final, or the first or final words depending on the version (cf. Daneman and Carpenter, 1980; Rönnberg et al., 1989; Waters and Caplan, 1996; McCabe et al., 2010). As previously noted, the reading span, as opposed to a listening span version, is preferable when speech is involved in order to avoid a confound with hearing acuity or stimulus clarity.

We earlier cited the claim by Caplan and Waters (1999), based on their work and the work of others, that working memory, at least as tested with the reading span task of Daneman and Carpenter (1980) and its variants, does not constrain, or by inference carry, on-line sentence comprehension. In contrast, the well-known meta-analysis by Daneman and Merikle (1996) showed reading span scores to reliably predict performance on a number of language comprehension and language memory tasks.

In addition to mixed findings in experimental studies relating reading spans to efficacy in language comprehension (see, the review in Wingfield et al., 1998) there is a similar case for the ability of working memory span as measured by reading span, as a predictor of perception of speech in noise or with reduced hearing acuity (cf. Akeroyd, 2008; Schoof and Rosen, 2014; Füllgrabe et al., 2015).

It is possible that the mixed findings in studies using the reading span as a measure of verbal working memory may lie in the intentional complexity of the reading span task itself, with this complexity allowing task demands or nuances of the instructions to affect the sensitivity of the span scores across different experiments. When one considers the reading span task it can be seen that there is an opportunity for a trade-off on the part of the reader or listener between recalling the sentence-final or sentence-initial words versus processing efficiency on the sentence comprehension component of the task. Indeed, individual differences in strategy use and session-to-session variability has been shown to occur in even less complex memory tasks (e.g., Logie et al., 1996).

Waters and Caplan (1996) recognized that the reading span task, because it involves both storage and processing components, is a better measure of working memory than a simple span test that has only a storage component. The task also has face validity as both the reading span task and language comprehension require temporary storage of verbal material along with ongoing syntactic and semantic computations. As Waters and Caplan note, this complexity of the Daneman and Carpenter (1980) reading span task focuses solely on the storage component of the task (recalling the sentence final words as the span measure) but not the efficiency with which the sentence comprehension component is conducted. To overcome this limitation they suggest a more valid measure might be represented by an index that takes into account sentence comprehension accuracy, the number of sentence final words that can be recalled, and as a measure of efficiency at sentence processing, response times to the sentence judgments. Represented as a z-score they show this composite measure to have better test-retest reliability than the original Daneman and Carpenter (1980) span test.

An additional criticism of the Daneman and Carpenter (1980) span test is that participants always know in advance that they will be asked to recall the last word of each of the sentences. That knowledge might lead to development of processing strategies by the participant. To overcome this issue Rönnberg et al. (1989) developed a span task that uses a post-cueing method in which the participant reads the stimulus sentences without knowing in advance whether they will be asked to recall the first or the final word of each sentence. This instruction is given after a sentence set has been presented.

In these regards, we suggest that a large-scale meta-analysis of studies compare and contrast findings using extant variations of the reading span task. Such an analysis should include relative strengths in terms of test-retest reliability where available.

The above discussion has focused more on the reading span as a measure of working memory capacity than on the memory systems that may be involved in speech comprehension at the sentence level. On the one hand, our discussion of “good enough” sentence processing suggests that an abstract representation of sentence meaning is formed as a sentence is being heard. On the other hand, our ability to “replay” the sensory input to retroactively repair an initial misanalysis of a garden-path sentence implies the support of a briefly sustained veridical trace of the input.

This apparent paradox was recognized by Potter (1993; Potter and Lombardi, 1990), who proposed that as a sentence is heard, both a verbatim trace of the spoken input and a semantic abstraction are concurrently formed and briefly stored in memory. Depending on the momentary needs of the listener or complexity of the speech materials, the individual might rely more or less heavily on the transient verbatim trace, whether this is thought of as a phonological, articulatory, or echoic store. In everyday listening the default mode may be reliance on the abstracted semantic trace for constructing narrative coherence, with the concurrently available verbatim trace accessible for a brief period if needed for specific task requirements or if access to the original input is needed in order to rescue an initial processing error. In the case of understanding meaningful speech, such a model might account at least in part for many of the paradoxes outlined above.

## Resource-Limited versus Data-Limited Processes

In performing a complex cognitive task one would expect that, at least to some limit, the level of performance will improve with the amount of effort (resources) given to that task. This refers to a task that is “resource-limited”: the upper limits on performance will be set only by the amount of resources one is willing, or able, to apply to it (Norman and Bobrow, 1975). In cases of degraded input, performance can often be improved with additional effort. There are other cases where the stimuli are of such poor quality that no amount of effort or allocation of resources will improve the level of performance. In such cases, when the upper limit on performance is determined by the limited quality of the stimulus, the task can be referred to as data-limited (Norman and Bobrow, 1975). Most tasks, even ones with a poor quality stimulus, are resource-limited up to some point where one’s performance is limited only by the amount of resources one is willing to devote to it. It is only beyond this point that one can say that the task is data-limited. Although questions have arisen about distinguishing between a data-limited transition and possible constraints of a ceiling effect (Norman and Bobrow, 1975; Kantowitz and Knight, 1976). Norman and Brobrow’s(1975) conceptualization is a descriptively important one.

Within the context of what Norman and Bobrow (1975) would call the resource-limited range, one can describe three “zones” of listening conditions: (1) effortless listening, where working memory resources are not drained by perceptual processing demands, (2) effortful but successful listening where errors will occur unless resources can be reallocated from other tasks, and (3) effortful but error-prone listening which is not yet data-limited, but where there are insufficient or non-optimally allocated resources (see Schneider and Pichora-Fuller, 2000; Pichora-Fuller, 2003, for discussions). Poor-hearing older adults would reach these points of effortful listening with higher sound levels than those with better hearing, and they would be reached sooner for more complex speech materials than simpler materials.

Although traditionally theorists have focused on just one direction of activity, whether on limited resources constraining perceptual effectiveness (Kahneman, 1973; de Fockert et al., 2001; Lavie et al., 2004) or perceptual effort reducing higher-level cognitive effectiveness (Rabbitt, 1968, 1991; Dickinson and Rabbitt, 1991; Murphy et al., 2000) one can postulate a single interactive dynamic which may operate in both directions: limited resources may impede successful perception when the quality of the sensory information requires perceptual effort for success, while successful perception in the context of a degraded stimulus or a hearing loss may draw on resources that might otherwise be available for downstream cognitive operations. These notions fit acceptably within the ELU model and it is hoped that they are more fully developed in future versions of the model.

## Conclusion

The ELU model can fairly be represented as a work in progress with many gaps to be filled. The model nevertheless serves as a useful framework for thinking critically about language understanding, especially under difficult listening conditions. That is, a model has value not only when it answers all of our questions, accounts for extant data, and makes specific predictions for experiments yet to be conducted. A model also has value when close scrutiny highlights what we know and what we do not know; the broader the sweep of the model the more this is likely to be so.

Our goal in this discussion has been to point to places in the model where there are gaps that are yet to be filled and where the model could be productively expanded. In doing so we acknowledge that the ELU model represents a unique attempt to formulate a unifying framework to describe sensory-cognitive interactions especially under difficult listening conditions.

An important feature in the development of the ELU model has been a shared focus both on theory and on the practical implications of cognitive resources in remediation in the case of hearing loss (e.g., Rudner and Lunner, 2013). The effectiveness of the rapid development of sophisticated signal processing algorithms, whether in traditional hearing aids or in cochlear implants, must take into account the cognitive supports and cognitive constraints of the user, especially, we suggest, in the case of the older listener. The integrative approach of the ELU model offers an ideally suited framework on which to carry continued research on this critical interaction.

### Conflict of Interest Statement

The authors declare that the research was conducted in the absence of any commercial or financial relationships that could be construed as a potential conflict of interest.
